# The prevalence of giant cell arteritis and polymyalgia rheumatica in a UK primary care population

**DOI:** 10.1186/s12891-016-1127-3

**Published:** 2016-07-15

**Authors:** Max Yates, Karly Graham, Richard Arthur Watts, Alexander James MacGregor

**Affiliations:** Department of Rheumatology, Norfolk and Norwich University Hospital, Colney Lane, Norwich, NR4 7UY UK; Norwich Medical School, Bob Champion Research and Education Building, University of East Anglia, Norwich, Norfolk NR4 7UQ UK

## Abstract

**Background:**

To update community-based prevalence values for Polymyalgia Rheumatic (PMR) and Giant Cell Arteritis (GCA) using case record review supplemented by population survey and subsequent clinical review.

**Methods:**

Clinical data were obtained from case records of a large primary care practice in Norfolk, UK and reviewed for diagnoses of GCA and PMR. In addition postal survey was carried out to capture potentially undiagnosed cases within the practice population. Those screening positive for potential diagnoses of GCA and PMR were invited for clinical review. A cumulative prevalence estimate was subsequently calculated on those diagnosed within the GP practice and subsequently on those fulfilling the various published classification criteria sets. The date of the database lock and mail merge was March 2013.

**Results:**

Through detailed systematic review of 5,159 GP case records, 21 patients had a recorded diagnosis of GCA and 117 had PMR.No new cases were identified among 2,227 completed questionnaires returned from the population survey of a sample of 4,728. The resulting cumulative prevalence estimate in those aged ≥ 55 years meeting the ACR classification criteria set for GCA was 0.25 % (95 % CI 0.11 to 0.39 %) and for five published criteria sets for PMR ranged from 0.91 to 1.53 % (95 % CI ranges 0.65 %, 1.87 %). The prevalence of both conditions was higher in women than in men and in older age groups.

**Conclusion:**

This study provides the first UK prevalence estimate of GCA and PMR in over 30 years and is the first to apply classification criteria sets.

## Background

Giant Cell Arteritis (GCA) is a vasculitis that predominantly affects large arteries and has substantial morbidity with permanent visual loss occurring in up to 35 % of patients [1, 2]. Polymyalgia rheumatica (PMR) is an inflammatory condition of unknown aetiology causing pain and stiffness in the shoulder and hip girdle [3, 4]. Both conditions overlap and are rarely diagnosed in individuals under 50 years of age, with older age being part of many of the classification criteria sets for both diseases.

Their prevalence is poorly reported globally. The only prevalence study of GCA and PMR from the UK to date was conducted in 1985 by Kyle et al. in a single Cambridgeshire practice [5] and has not been repeated. The resultant prevalence estimate of GCA was 1.23 % for those aged ≥ 65 years [5] and for GCA combined with PMR was 3.5 % [5]. The Kyle et al. study pre-dates the current ACR classification criteria set and failed to include participants younger than 65 years. Both conditions are commoner in older age groups and the life-expectancy of the UK population is increasing [6]. More recent data from Europe and the USA have given widely varied prevalence estimates, with an over 30-fold discrepancy (see Table 1). Data from the Mayo clinic from all incident cases of GCA from Olmsted County (years 1950 to 1999), revealed 173 cases of GCA in those aged ≥ 50 years. This resulted in a prevalence estimate for GCA of 0.23 % and for PMR of 0.74 % (95 % CI 0.67 to 0.81) [7–9]. Data from Germany from a questionnaire sent to hospital departments and insurance companies in 2006 revealed a prevalence for GCA of 0.04 % (95 % CI 0.04 to 0.05) [10].

**Table 1:** Published estimates for GCA and PMR prevalence

Author	Country; survey type; age threshold	GCA and PMR prevalence	Classification
Kyle et al. 1985 [5]	UK; GP practice based survey; ≥ 65 years.	GCA: 1.23 % (95 % CI 0.38 to 2.08) PMR: 1.69 % (95 % CI 0.70 to 2.68)	GCA: Jones and Hazleman PMR: Jones and Hazleman
Koboyashi et al. 2003 [20]	Japan; Hospital only treated patients in 1997; ≥50 years	GCA: 0.002 %	GCA: ACR 1990
Lawrence et al. 2008 [7] Salvarni et al., 1999 [8]. Doran et al. 2002 [9].	USA; Olmsted County survey of cumulative incidence 1950–1999; ≥50 years	GCA: 0.28 % (95 % CI, 0.19–0.27) - PMR: 0.74 % (95 % CI, 0.67–0.81)	GCA: ACR 1990 PMR: Doran et al.
Mohammad et al. 2011 [21]	Skåne,Sweden; survey of cumulative incidence 1997 – 2010; ≥50 years	GCA: 0.11 % (95 % CI, 0.10–0.12).	GCA: Temporal artery biopsy positive only
Herlyn et al. 2014 [10]	Germany; Survey of hospitals, private physicians and insurance companies in 2006; ≥50 years	GCA: 0.04 % (95 % CI 0.04 to 0.05)	GCA: ACR 1990
Salaffi et al. 2005 [24]	Italian MAPPING study; population survey; age ≥65 years	PMR: 0.37 % (95 % CI 0.29–0.44)	PMR: Bird et al.
Bernatsky et al. 2009 [23]	Canada; hospital record survey, cumulative incidence 1995–2006; ≥50 years	PMR: 0.64 % (urban); 0.86 % (rural)	PMR: Physician billing twice within 2 months or stated on hospital discharge.
Pamuk et al. 2009 [19]	Turkey. Single rheumatology department in a tertiary referral centre, cumulative incidence, ≥50 years	PMR and GCA: 0.02 %	GCA: ACR 1990 PMR: Chuang et al.

Accurate prevalence estimates for GCA and PMR enable the burden of disease and associated costs to the health service to be calculated and development of appropriate services to manage this group of patients. The aim of this study was to provide contemporary estimates of prevalence in a representative UK community sample using current classification criteria sets.

## Methods

### Design

General Practice case record review, supplemented by subsequent postal survey and sampling involving clinical examination by a rheumatologist.

### Setting

Drayton and St Faith’s Medical Practice, a dispensing medical practice situated to the north-west of the city of Norwich, UK. The practice includes urban and rural areas, residential suburbs and extends into outlying villages. The practice provides care for approximately 13,000 individuals. In the UK it is usual for patients to register with their local practice and this practice falls within the catchment area of Broadland district in the county of Norfolk. The five electoral wards covered by the practice have a combined population (from the 2011 Census) of 15,102 of whom 5,108 (34 %) were aged ≥55 years and 2,983 (20 %) were aged ≥65 years. The practice is representative of the Norfolk population in terms of ethnicity, gender ratio and age structure (36 % of the Norfolk population are aged ≥55 years).

### Database search

Cases of GCA and PMR were identified through a GP database review. All individuals aged ≥ 55 years were included. An age cut-off of 55 years was used for pragmatic reasons as cases of GCA and PMR are rare at younger ages. Read code analysis (GCA: G755., G7550, G7551, G7552, G755z and PMR: N20.., N200.) and key word searches (*polymyalgia rheumatica, giant cell arteritis, temporal arteritis*) were performed to interrogate the electronic GP register. To satisfy a GP diagnosis of either GCA or PMR, patients needed to have received treatment with glucocorticoids *AND* a diagnosis that was not later refuted. Those whose diagnosis was later refuted were considered not to have the conditions. The search, and subsequent database lock and mail merge was carried out on the 8^th^ March 2013.

### Case classification

#### GCA

To be classified as GCA, cases were required to fulfil the 1990 ACR criteria set (at least 3 from the following 5): 1. Age at disease onset > =50 years *(Development of symptoms or findings beginning at age 50 or older);* 2. New headache *(New onset of or new type of localised pain in the head);* 3. Temporal artery abnormality *(Temporal artery tenderness to palpation or decreased pulsation, unrelated to arteriosclerosis of cervical arteries);* 4. Elevated erythrocyte sedimentation rate *(Erythrocyte sedimentation rate > =50 mm/h by the Westergren method);* 5. Abnormal artery biopsy *(Biopsy specimen with artery showing vasculitis characterised by a predominance of mononuclear cell infiltration or granulomatous inflammation, usually with multinucleated giant cells)* [11].

#### PMR

There are several classification criteria sets for PMR [12–17] sharing some common features (Table 2). Key information (clinical features and results of inflammatory markers) were extracted from patients’ case records to enable classification by five distinct criteria sets: Bird [12]; Chuang [13]; Healey [14]; Jones and Hazleman [15], and Doran [9]. The newer criteria published in 2012 have a point scoring system and incorporate the results of either rheumatoid factor or anti-CCP antibody [17]. However, in the UK, rheumatoid factor (RhF) and anti-CCP antibody (ACPA) are not uniformly requested in patients with possible PMR [18] and these criteria were not used.

**Table 2:** Classification Criteria for Polymyalgia Rheumatica

Authors & Year	Proposed Criteria	Requirement for Classification
Bird et al. (1979)	Age ≥65 years Bilateral shoulder pain and stiffness; acute or subacute onset (<2 weeks); morning stiffness >1 h depression and/or weight loss; bilateral tenderness in upper arm muscles ESR >40 mm/h	Any three, or any one plus temporal artery abnormality (including decreased pulsation, tenderness, beading or bruit).
Jones and Hazelman (1981)	Shoulder and pelvic girdle pain; morning stiffness >1 h; exclusion of rheumatoid arthritis or other inflammatory arthropathy, myopathy, malignancy ESR >30 mm/h or CRP >6 mg/L Rapid response to corticosteroids	All criteria must be met
Chuang et al. (1982)	Age ≥50 years >1 month bilateral aching and stiffness of at least two of the following areas: Neck or torso, shoulders or proximal arms, hips or proximal thighs; exclusion of other causes ESR >40 mm/h	All criteria must be met
Healey and Wilske (1984)	Age ≥ 50 years >1 month of neck, shoulder, or pelvic girdle pain (any two areas); morning stiffness >1 h; exclusion of other diagnoses ESR ≥40 mm/h Rapid response to daily, low-dose steroid therapy (i.e., prednisolone ≤20 mg)	All criteria must be met
Doran et al. (2002)	Age ≥ 50 years; Bilateral aching and morning stiffness (lasting ≥ 30 min) persisting for at least 1 month and involving 2 of the following areas: neck or torso, shoulders or proximal regions of the arms, and hips or proximal aspects of the thighs ESR > 40 mm/h OR rapid response to corticosteroids	All criteria must be met
Dasgupta et al. (2012)	Morning stiffness ≥ 45 min (2 points); Hip pain, limited range of movement (1 point) Absence of other joint pain (1 point) Normal RhF or ACPA (2 points) Ultrasound criteria: at least 1 shoulder with subdeltoid bursitis and/or biceps tenosynovitis and/or glenohumeral synovitis AND at least 1 hip with synovitis and/or trochanteric bursitis (1 point); both shoulders with subdeltoid bursitis, bicep tenosynovitis or glenohumeral synovitis (1 point)	All patients must be: Age ≥50 years, have bilateral shoulder aching and abnormal ESR/CRP Scoring algorithm without ultrasound score of 4 needed – with ultrasound score of 5 needed

#### Sampling frame

To capture potentially undiagnosed cases, the entire practice population aged ≥ 55 years was surveyed using a questionnaire specifically designed to detect cardinal features of GCA and PMR. Patients were excluded if they had dementia, a terminal illness, were living in a nursing home or had previously informed the practice they did not wish to take part in research. Non-responders were sent a reminder invitation and questionnaire after three months.

#### Questionnaire development

No questionnaire currently exists for classifying people with GCA or PMR for diagnostic purposes. In order to capture undiagnosed cases, we required an instrument sensitive to the detection of the broadest possible range of symptoms of PMR and GCA. The diagnosis could be confirmed or refuted by later applying clinical judgement during a patient visit. A new questionnaire was developed by adapting the 1985 questionnaire used by Kyle et al. [5], to include the 1990 ACR criteria set [11] and informed by a review of the nature and frequency of symptoms reported from GCA and PMR cohorts published in the literature. This questionnaire captured the following symptoms: headache, visual disturbance, scalp tenderness, symptoms suggestive of jaw claudication, shoulder pain and stiffness, myalgia, weight loss, general malaise, and important potential discriminative symptoms of migraine. The questionnaire also asked participants whether they had ever been diagnosed by their GP with either GCA or PMR. Its face validity was confirmed in a hospital sample of ten patients with GCA and or PMR, and among three rheumatologists and two rheumatology nurse practitioners specialising in vasculitis.

#### Clinical review of potential cases

Based on their questionnaire responses, all participants who answered positively to questions on jaw claudication AND scalp tenderness AND visual disturbance, with or without the presence of headache and who were not already known to the practice as having a diagnosis of GCA, were invited for clinical review by a rheumatologist. This group was considered as having a high likelihood of having a diagnosis of GCA. Those answering positively to one or more questions were considered at intermediate likelihood of having GCA or PMR and a random sample (*Excel Microsoft Corporation* random number generator) of these was invited for clinical review. Those included in the random sample would be expected to contain potentially undiagnosed cases of PMR or GCA.

### Sample size calculation

A sample size of 4,000 allowed the detection of an anticipated prevalence of 0.3 % for GCA with 95 % confidence interval ranging from 0.13 to 0.47 %. A greater level of precision would be possible for PMR as it is more common than GCA.

### Analysis

Minimum cumulative prevalence estimates were calculated for both diseases. This method assumes that all known cases are identified, those who have died within the population are excluded and that non-responders to the survey do not have the disease of interest. This method also takes account of any deaths that have occurred in both numerator and denominator populations, unlike cumulative incidence methods. Cumulative prevalence estimates were expressed as a proportion; confidence intervals were calculated based on the Poisson distribution.

## Results

### Database search

**Fig. 1:**
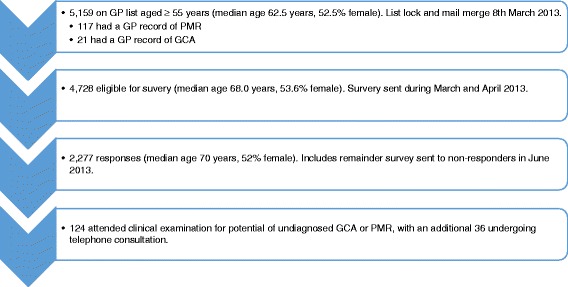
Flowchart of sampling method to generate cumulative prevalence estimates

A total of 5,159 patients registered with the practice were aged ≥55 years with a median age of 68 years. Of these 2,706 (52.5 %) were female. Searching the electronic register identified 21 with GP-recorded GCA and 117 with GP-recorded PMR (Fig. 1 shows a flowchart for the sampling method).

### Survey

Subsequently, questionnaires were sent to 4,728 eligible patients of the 5,159 registered with the practice to ascertain any missed diagnoses of GCA and PMR not recorded in the GP electronic register. A total of 2,277 (48 % response rate) questionnaires were returned of which 97.2 % had complete data. The respondents had a median age of 70 years and 52 % were female. The age and sex distribution of the non-responders was similar to that of the responders (Table 3).

**Table 3:** Characteristics of survey responders

Characteristics	Total Population	Survey Pool	Survey Responders	PMR		GCA	
				Self –reported	Confirmed by GP	Self –reported	Confirmed by GP
Number ≥55 years	5,159	4,728	2,277	83	73	15	9
Age in years (median)	62.5	68.0	70.0	75.6	75.8	72.1	73.9
Percentage Female (%)	52.2	53.6	52.0	70.0	67.4	78.6	71.5
Clinical features reported on questionnaire							
Headache (%)			4.7	8.4	9.5	31.3	55.6
Jaw Pain (%)			7.5	20.5	19.2	31.3	44.4
Visual Disturbance (%)			19.8	22.9	24.7	37.5	44.4
Scalp Tenderness (%)			7.8	15.7	6.2	56.3	88.9
Shoulder Pain (%)			24.9	67.5	67.1	50	77.8
Myalgia (%)			18.8	68.7	67.1	43.8	55.6
Unexplained Weight loss (%)			4.2	12.1	11	31.3	55.6
Migraine with aura (%)			15	14.6	16.7	18.8	11.1
No Symptoms (%)			55.8	20.5	21.9	18.8	0

### Clinical review of questionnaire respondents

A total of 15 individuals reported a diagnosis of GCA, 9 of whom were confirmed by the practice record. All 9 GP-recorded cases of GCA fulfilled the 1990 ACR classification criteria. In all cases the diagnoses were confirmed after referral to secondary care specialists including rheumatologists, ophthalmologists, geriatricians and neurologists. These subjects were not invited for further clinical review.

Among the remaining 2,268 respondents who were not already known from the GP-record as having a diagnosis of GCA or PMR, 31 reported the triad of visual disturbance, scalp tenderness and symptoms suggestive of jaw claudication. All these were contacted and, after clinical review, none were thought to have GCA. At least one symptom was reported in 1,007. Of these a sample of 93 were reviewed, 25 had reported symptoms suggestive of PMR without key features of GCA (i.e., lack of headache, jaw claudication, visual disturbance or scalp tenderness). After clinical review none were thought to have undiagnosed PMR nor GCA. All those invited attended for assessment. Table 3 lists the clinical characteristics of the survey responders.

Of the 83 who reported a diagnosis of PMR on the questionnaire, 73 cases were confirmed to have the condition by the practice. The diagnoses of the two respondents with both PMR and GCA were confirmed by practice records.

Individuals reporting diagnoses of either GCA (*n* = 6) or PMR (*n* = 10) but without confirmation on the GP record were contacted by telephone. The discrepancy was found to be either due to participants mistaking arteritis for arthritis on the questionnaire, or to the diagnoses being refuted by the GP later and withdrawn.

### Case classification

Of the 21 cases identified in the GP record with GCA, a total of 13 fulfilled the 1990 ACR classification criteria set based on the information included in the GP record, with a median age of 70 years at diagnosis and a mean ESR of 74 mm/h. The remaining eight failed to fulfil classification criteria based on their inflammatory marker results and the absence of typical histological findings on temporal artery biopsy; of these, six had insufficient diagnostic information on their records having changed practice.

There are five commonly used classification criteria sets for PMR used in the previous published estimates. All rely on the combination of typical clinical features, age and elevated inflammatory markers but the cut-points for these vary (Table 2).

Of the 117 cases identified in the GP records with PMR, 73 (71 %) were female and the median age of 70 years. Inflammatory marker results, prior to steroid initiation, were available for 100 patients (96 with ESR; mean 45 mm/h, 33 with CRP; mean 50 mg/dL and 29 had both). Based on the data from these 100 individuals, the estimated proportion of those satisfying the different criteria sets are: 47 % (Bird); 62 % (Chuang); 62 % (Healey); 62 % (Doran); and 79 % (Jones and Hazleman). The majority (71 %) of PMR cases were managed exclusively in primary care and no further information was available for more detailed classification.

### Prevalence estimates

Based on the cases which could be identified reliably through GP record review (namely those who had treatment with glucocorticoids *and in whom the* diagnosis that was not later refuted), the minimum cumulative prevalence estimate for GP-diagnosed cases of GCA was 0.41 % (95 % CI 0.23 %, 0.58 %) and for PMR was 2.27 % (95 % CI 1.86 %, 2.67 %) (Table 4). The table also shows age and sex specific cumulative prevalence for both conditions.

**Table 4:** Cumulative prevalence estimate by sex and age bands based on GP records

Age	N^a^	Female	Male	PMR		Female		Male		GCA		Female		Male	
				N	Pr (95 % CI)	N	Pr (95 % CI)	N	Pr (95 % CI)	N	Pr (95 % CI)	N	Pr (95 % CI)	N	Pr (95 % CI)
55–60	978	500	478	1	0.10 (0.00, 0.30)	1	0.20 (0.00, 0.59)	0		1	0.10 (0.00, 0.30)	0		1	0.21 (0.00, 0.62)
60–69	1902	989	913	20	1.05 (0.59, 1.51)	15	1.52 (0.76, 2.28)	5	0.55 (0.07, 1.03)	3	0.16 (0.00, 0.34)	3	0.30 (0.00, 0.65)	0	
70–79	1412	700	712	49	3.47 (2.52, 4.43)	33	4.71 (3.14, 6.28)	16	2.25 (1.16, 3.33)	6	0.43 (0.09, 0.76)	4	0.57 (0.01, 1.13)	2	0.28 (0.00, 0.67)
80–89	695	395	300	40	5.76 (4.02, 7.49)	20	5.06 (2.90, 7.22)	20	6.67 (3.84, 9.49)	9	1.30 (0.45, 2.14)	5	1.27 (0.16, 2.37)	4	1.33 (0.04, 2.63)
90–100	166	116	50	7	4.22 (1.16, 7.27)	5	4.31 (0.62, 8.01)	2	4.00 (0.00, 9.43)	2	1.72 (0.00, 4.09)	2	1.72 (0.00, 4.09)	0	
100+	6	6	0	0		N/A		N/A		0		N/A		N/A	
Total	5159	2706	2453	117	2.27 (1.86, 2.67)	74	2.74 (21.12, 3.35)	43	1.75 (1.23, 2.27)	21	0.41 % (0.23, 0.58)	14	0.52 (0.25, 0.79)	7	0.29 (0.07, 0.50)

*Pr* cumulative prevalence

^a^N total number of people registered with the practice

The cumulative prevalence of GCA for those in whom it was possible to apply the 1990 ACR classification criteria retrospectively was 0.25 % (95 % CI 0.11 to 0.39). Applying the various classification criteria retrospectively to GP-diagnosed cases of PMR (Table 5) gave the following cumulative prevalence estimates: Bird criteria 0.91 % (95 % CI 0.65 to 1.17); Chuang, Healey and Doran criteria 1.20 % (95 % CI 0.90 to 1.50); and Jones and Hazleman criteria 1.53 % (95 % CI 1.20 to 1.87).

**Table 5:** Cumulative prevalence estimates for cases which satisfy classification criteria applied to GP record data

Criteria set	All		Female		Male	
	N	Pr (95 % CI)	N	Pr (95 % CI)	N	Pr (95 % CI)
GCA (21 cases reported in GP records)						
Hunder	13	0.25 (0.11, 0.39)	9	0.33 (0.12, 0.55)	4	0.16 (0.00, 0.32)
PMR (117 cases reported in GP records)						
Bird	47	0.91 (0.65, 1.17)	28	1.04 (0.65, 1.42)	19	0.78 (0.43, 1.12)
Healey; Chuang; Doran	62	1.20 (0.90, 1.50)	39	1.44 (0.99, 1.89)	23	0.94 (0.56, 1.32)
Jones and Hazleman	79	1.53 (1.20, 1.87)	49	1.81 (1.31, 2.31)	30	1.22 (0.79, 1.66)

N total number of people identified in the practice. Pr: cumulative prevalence based on a total population of 5,159 (2,706 female, 2,453 male). Inflammatory markers were available in 100 out of 117 GP record cases (of which 96 had ESR and 33 CRP with overlap of 29 cases with both CRP and ESR results at time of diagnosis)

## Discussion

### Principal findings

These are the first prevalence estimates for GCA and PMR from a community sample in the UK for over 30 years and the first to apply contemporary classification criteria. Our study was confined to single practice in Norfolk, UK and faced challenges in applying existing classification criteria retrospectively. However, a major strength of the study was that every effort has been made to ascertain cases of GCA and PMR which may have not been known to the GP practice.

This study’s prevalence estimate for GCA is lower than that reported in the 1985 UK community-based study by Kyle et al. which examined 650 individuals aged over 65 years from a single GP practice, screened by questionnaire and those with possible GCA or PMR assessed by a rheumatologist. After case record review, eight cases fulfilled the existing criteria giving a prevalence estimate for GCA of 1.23 % (95 % CI 0.38 to 2.08) [5], almost 5-fold higher than the present study estimate. This excess is likely only to be partly explained by the older age group (age > 65 vs > 55) and their use of a lower ESR cut-off level (30 mm/h vs 50 mm/h). The UK prevalence ranges for PMR estimated in the present survey (0.91 to 1.53 %) is similar to that reported by Kyle et al. (1.69 % (95 % CI 0.70 to 2.68)) [5].

The wide variation in prevalence estimates for GCA and PMR in more contemporary studies (Table 1) may be due to differences in the methods used for selection of cases; for example a number ascertained cases only through secondary care or even tertiary referral centres [19–21]. Definitions of disease also vary. Applying classification criteria sets rigorously in population studies of these relatively rare diseases is logistically difficult as it requires patient contact, laboratory tests results including inflammatory markers and, for GCA, the results of temporal artery biopsy. For these reasons contemporary studies of GCA prevalence have largely relied on a hospital-based assessments.

Data from Olmsted County Minnesota from the Rochester epidemiology project have been used to calculate prevalence based on cumulative incidence rates. GCA cases (*n* = 173) were recorded in Olmsted County over a 50 year period (1950 to 1999) and PMR cases recorded between 1970 and 1999, giving estimates of 0.23 and 0.74 % respectively. The estimates of incidence were age-adjusted to the 1980 US white population. In 1980 in Olmsted County there were 92,006 individuals (of whom approximately 10 % were aged ≥50 years) recorded in the US census and this rose to 144,248 (of who 45,325 (31 %) were aged ≥50 years, 34,135 (24 %) were aged ≥55 years or older and 25,037 (17 %) were aged ≥ 60 years) in 2010. Since the incidence of both conditions increases with age it is likely that the resultant derived prevalence estimates will underestimate the true prevalence when age-adjusting to a 1980 US average.

The lowest contemporary European estimate of GCA prevalence (0.04 %) was generated by a 2014 German study in which diagnoses were ascertained from hospital insurance records. Record data have also been used in two studies of GCA which have examined a UK GP electronic database, known as the Clinical Practice Research Datalink (CPRD), but did not attempt to apply classification criteria nor estimate prevalence [6, 22]. The study by Petri et al. reported GCA incidence but estimated prevalence for those aged ≥50 years as 0.33 % (personal communication). Comparing our results to those of Petri, we re-caculated the cumulative prevalence based on the practice population aged ≥50 years. Since no persons in the practice aged between 50 and 55 years had been diagnosed with GCA this gave a cumulative prevalence of 0.34 % (95 % CI 0.20, 0.49), similar to that found using the CPRD.

Studies of PMR have used a variety of classification methods with a number developing study-specific criteria for case recognition. For example, Bernatsky et al. [23] identified cases through billing and hospitalisation; Doran et al. [9] developed their own classification system. The present study shows that differences between existing classification sets, when applied to the same population, may account for an almost two-fold difference in prevalence estimates. The use of Bird’s criteria set resulted in the lowest estimate. The Italian MAPPING study also used these criteria and generated the lowest European estimate to date for PMR prevalence [24].

In addition, four studies used cumulative incidence to derive a prevalence estimate (Table 1). This method assumes that the case mortality rate is comparable to that of the general population. A number of studies have shown GCA to be associated with an excess mortality risk, particularly in the first two years after disease onset [25, 26]. This may upwardly bias the prevalence estimates in these studies. The mortality risk associated with PMR is not firmly established. Left censoring in short-term cumulative incidence studies (for example [19, 23]) might lead to an underestimate of prevalence.

### Study limitations

Our estimates can only be applied to individuals aged ≥55 years. It should be noted that in our sample no cases of GCA and only 5 % of GP-recorded cases of PMR developed before the age of 55. The population was White British and was restricted to one general practice partnership in Norfolk and therefore the generalisability of our results to more ethnically diverse regions of the country or other countries is relatively limited. The percentage of people aged ≥55 years in Norfolk is 36 %. This is higher than the England and Wales figure of 29 %. Given the older age of the Norfolk population it is likely that both incidence and prevalence estimates for GCA and PMR will be higher than in the UK population as a whole. However this region of the UK has provided epidemiological data for the Norfolk Arthritis Register (NOAR) cohort and provided estimates for the primary systemic vasculitides [27, 28].

Every effort was made to enrich the available clinical data through both direct record review and questionnaire sampling, supplemented by clinical assessment. While laboratory data were available in all cases of GP recorded GCA, the results of tests of inflammatory makers were missing in the clinical record in 15 % of those assigned a GP diagnosis of PMR. Incomplete GP records prevented us from confirming a diagnosis of PMR where blood test results prior to the year 2000 could not be retrieved electronically. If the analysis is confined only to those who responded to the questionnaire, prevalence estimates for GCA would be 0.19 % (95 % CI 0.07 % to 0.32 %) and for PMR would be 1.54 % (95 % 1.19 % to 1.90 %).

In designing the study, we were aware that no validated questionnaire exists for classification or diagnosis of either GCA or PMR. A concern was that cases might exist in the community, which had not been identified by their GP. In the 1985 study by Kyle et al., one case of GCA was identified which had been unknown to their GP. Historical diagnoses are difficult to capture through questionnaires, particularly in diseases that remit with treatment. Cases that had entered remission before 2000 who were also not captured on the GP records may have been difficult to detect in this study. However, by using the combined approaches for case ascertainment in this study, we feel it unlikely that we failed to capture cases of undiagnosed or remitted disease. Our results suggest that GP-recorded diagnoses are likely to encompass all cases within a population, although not all will fulfil classification criteria sets.

## Conclusions

In conclusion we have provided the first contemporary data on GCA and PMR in the UK for 30 years. These conditions have been confirmed to be, respectively, the most common forms of vasculitis and inflammatory rheumatic disease in the UK and will become commoner as the population ages since their peak incidence occurs in individuals older than 70 years. Resources need to be directed at managing these conditions and to understand their aetiology with the aim of improving outcomes.

## Abbreviations

ACPA, anti-citrullinated protein antibody; CRP, C-reactive protein; ESR, erythrocyte sedimentation rate; GCA, giant cell arteritis; GP, general practitioner; PMR, polymyalgia rheumatica; RhF, rheumatoid factor; UK, United Kingdom
